# 

**Published:** 1868-09-15

**Authors:** 


### CHICAGO MEDICAL JOURNAL.

J. ADAMS ALLEN, M.D., LL.D., Editor. WALTER HAY, A.M., M.D., Associate Editor.

All letters for the JOURNAL should be addressed to Dr. J. ADAMS ALLEN P. O. Box 1948, Chicago, Ill.

TERMS — $3 Per Annum, in Advance;

$4 if not paid before 1st of July, in each year. Single copies, 25 cents.

### BOOKS RECEIVED.

CRIMINAL ABORTION: Its Nature, Its Treatment, and Its Law. By Horatio R. Storer, M.D., LL.B., etc., etc., and Franklin Fiske Heard. Boston: Little, Brown and Company. 1868. Pp. 215.

VESICO-VAGINAL FISTULA, From Parturition and Other Causes; With Cases of Recto-Vaginal Fistula. By Thomas Addis Emmet, M.D., Surgeon-in-chief of the New York State Woman's Hospital, etc., etc. New York: William Wood and Company. 1868. Pp. 250.

ON DISEASES PECULIAR TO WOMEN; Including Displacements of the Uterus. By Hugh L. Hodge, M.D., Emeritus Professor of Obstetrics and Diseases of Women and Children, in the University of Pennsylvania. *Nullius addictus jurare in verba magistri.* With Illustrations. Second Edition, revised and enlarged. Philadelphia: Henry C. Lea. 1868. Pp. 531.

ATLAS OF VENEREAL DISEASES. By A. Cullerier, Surgeon, etc., etc., Paris. Translated by Freeman J. Bumstead, M.D., Professor, etc. Part IV. Philadelphia: Henry C. Lea. 1868.

TRANSACTIONS of the Medical Society of the State of Pennsylvania. Nineteenth Annual Session. 1868.

TRANSACTIONS of the Indiana State Medical Society. Eighteenth Annual Session. 1868.

OVARIOTOMY: A Paper read before the Ohio Med. Soc., Session 1868. By Alex. Dunlap, A.M., M.D., Springfield, Ohio.

TRANSACTIONS of the American Dental Association. Fifth Annual Meeting, held in Chicago, July 25th to July 28th, inclusive, 1865; and Sixth Annual Meeting, held in Boston, July 31st to August 7th, inclusive, 1866. Boston: Alfred Mudge and Company, 34 School street. 1868. Pp. 511.

